# Carotenoid Biofortification in Field-Grown Tomato Fruits by Early Inoculation with Arbuscular Mycorrhizal Fungi

**DOI:** 10.1021/acs.jafc.5c14198

**Published:** 2025-12-10

**Authors:** Javier Lidoy, Zhivko Minchev, Luis España-Luque, Ana M. Benítez-González, Andrea Ramos, Juan García, Estefanía Berrio, Olena Nesterenko, Pedro Díaz-Ortiz, Antonio J. Meléndez-Martínez, María J. Pozo, Juan A. López-Ráez

**Affiliations:** † Mycorrhiza Group, Department of Soil and Plant Microbiology, 73025 Estacion Experimental Del Zaidin (EEZ-CSIC), Calle Profesor Albareda 1, Granada 18008, Spain; ‡ Plant Immunity and Biochemistry Group, Department of Biology, Biochemistry and Natural Sciences, Universitat Jaume I, Castellón de la Plana 12071, Spain; § Food Color and Quality Laboratory, Facultad de Farmacia, 16778 Universidad de Sevilla, Sevilla 41012, Spain; ∥ SAT Hortoventas, Calle Estación s/n, Ventas de Zafarraya, Granada 18125, Spain

**Keywords:** AM symbiosis, biosynthesis, β-carotene, biostimulants, lycopene, *Solanum lycopersicum*, sustainable agriculture

## Abstract

Carotenoids are bioactive compounds with relevant health-promoting properties. Thus, a carotenoid-rich diet is essential for improving human health. Beneficial soil microorganisms are used in agriculture as biostimulants to promote plant growth and development and increase their tolerance/resistance to stress. However, their effects on fruit quality have been less studied. In the present study, we assess the impact of early inoculation of tomato seedlings with the arbuscular mycorrhizal (AM) fungus *Rhizophagus irregularis* on carotenoid content in fruits under real agronomic production settings. We show that early inoculation of seedlings with AM fungi provides long-lasting benefits that impact fruit quality, increasing the content of the carotenoids lycopene and β-carotene. We also show that this increase is related to transcriptional upregulation of key genes of their biosynthesis pathway. Our results show that AM fungi, commonly used as biostimulants in agriculture, can also be used as a sustainable strategy for carotenoid biofortification in tomato production systems, contributing to the production of healthy “functional products”.

## Graphical abstract

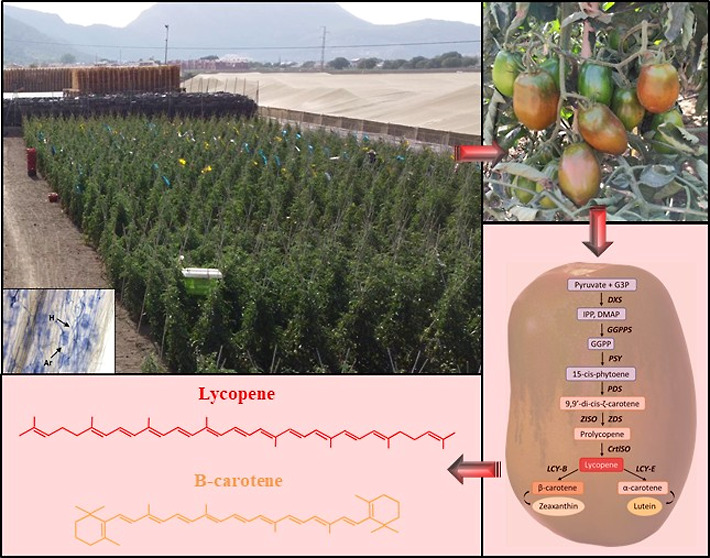

## 1 Introduction

Nutritional deficiency is becoming a universal problem, resulting in poor health and increasing rates of mortality and morbidity.^1^ Furthermore, micronutrient deficiency, also known as the hidden hunger, remains a significant problem in many developing countries.^2,3^ Therefore, there is an urgent need for sustainable production of healthy products to feed and care for the growing world population. In this regard, there is growing interest in the use of beneficial soil microorganisms, including arbuscular mycorrhizal (AM) fungi, *Trichoderma*, and plant growth promoting rhizobacteria, as an environmentally friendly alternative to synthetic fertilizers and pesticides. These microorganisms can be used as biostimulants to promote plant growth, stress tolerance, and defense responses.^4,5^ Fruits and vegetables constitute the major dietary sources of bioactive compounds with health-promoting properties, including antioxidant, anti-inflammatory, and antiaging capabilities. Carotenoids are among these bioactive compounds, which are being extensively studied in the context of sustainable food production for animal and human consumption.^6^ They are lipophilic metabolites widely present in nature, being produced by all photosynthetic organisms (plants, algae, and cyanobacteria) and also by some nonphotosynthetic microorganisms such as prokaryotes and fungi.

The first committed step of carotenoid biosynthesis is the formation of C40 phytoene from two molecules of geranylgeranyl diphosphate catalyzed by the enzyme phytoene synthase (PSY).^7,8^ Then, the colorless phytoene is converted through sequential desaturation and isomerization reactions by the enzymes phytoene desaturase (PDS), ζ-carotene desaturase (ZDS), and carotene isomerase (CrtISO) to lycopene, a red pigment. Further cyclization of the two ends of lycopene is the first branching step in the carotenoid pathway, giving rise to β-carotene by the action of lycopene β-cyclase (LCY-B) or α-carotene by the action of lycopene ε-cyclase (LCY-E).^7,8^ In plants, carotenoids act as photoprotectants against photo-oxidative stress and as pigments, imparting colors ranging from yellow to red.^1,3,9^ Carotenoids also have benefits for human health, mostly associated with their health-promoting biological actions (antioxidant, light absorption, anti-inflammatory, modulation of gene expression), contributing to reducing the risk of diseases such as cancer, cardiovascular, eye, skin, or metabolic pathologies.^10^ As most animals, humans cannot produce carotenoids and obtain them from their diet. Therefore, a carotenoid-rich diet is essential for better health. In this context, there are different strategies for supplementing and fortifying foods with carotenoids for nutritional, pharmaceutical, and even cosmetic purposes, such as the use of nutritional supplements and fortification.^2,10^ However, these strategies are not available to the entire population, especially in rural communities. A feasible strategy to address this problem may be crop biofortification; that is, the development of enriched plant varieties or methods for increasing the carotenoid content of plants. Current approaches for plant biofortification include conventional breeding, biotechnology, and agronomic management.^7^

Tomato (*Solanum lycopersicum*) is a staple food containing high levels of nutrients and bioactives, and its consumption is recommended by the World Health Organization (WHO). It is the most important fleshy fruit vegetable worldwide, with production (total fresh) exceeding 192 million tons in 2023 (FAO 2024). It is a low-calorie food and an important source of minerals (potassium and magnesium), vitamins, and antioxidants such as vitamin C, flavonoids, and carotenoids. The main colored carotenoids in tomato fruit are lycopene, accounting for about 90% of the pigments and responsible for the characteristic red color, and β-carotene, responsible for orangey colors and lutein, with a yellowish color.^8,11^ Lycopene is considered a powerful natural lipophilic antioxidant, which can help reduce oxidative damage and diminish the risk of developing degenerative pathologies. It has also been proposed that it has a beneficial role in the treatment of chronic diseases such as cancer, cardiovascular disease, and metabolic syndrome.^12^ β-Carotene is the primary dietary source of provitamin A (retinol) worldwide. Remarkably, vitamin A deficiency is a major problem in most developing countries, leading to blindness and development problems.^6,7^ Tomato fruit is also a good source of the colorless carotenoids phytoene and phytofluene, which have been shown to be major dietary compounds, bioavailable, and involved in biological actions that promote health.^13^ Thus, carotenoid biofortification in tomato fruits offers important benefits for human health, reducing the risk of diseases.^14^

Plant-associated microbes have been shown to affect plant secondary metabolism. Tomato is a mycotrophic plant, establishing mutualistic symbiosis with AM fungi. These fungi establish mutualistic associations with the roots of most terrestrial plants, including agricultural and horticultural crop species. This association dates back more than 450 million years, being crucial in plant evolution and an essential component of the plant microbiome.^15^ AM symbiosis has been shown to offer several benefits to tomato plants, including improved mineral acquisition and enhanced tolerance/resistance against abiotic and biotic stresses, usually associated with changes in secondary metabolism.^16,17^ Few studies also suggest that AM symbiosis can improve the nutritional value of tomato fruits under controlled conditions.^18–20^ However, results under field conditions are scarce and inconsistent. While few studies reported an increase of fruit carotenoid content in mycorrhizal plants,^21–23^ others did not find changes.^24–26^ To address this gap, we designed a solid real production system study, from commercial nursery to open-field production, to assess the impact of early inoculation of tomato plants with the AM fungus *Rhizophagus irregularis* on fruit carotenoid content (Figure 1). We hypothesized that early inoculation of rootstock seedlings with AM fungal spores at the nursery stage will provide long-lasting benefits by systemically impacting carotenoid metabolism in the fruits of the grafted variety. This knowledge will offer an important advance for future carotenoid biofortification strategies in tomato production.

**Figure 1:**
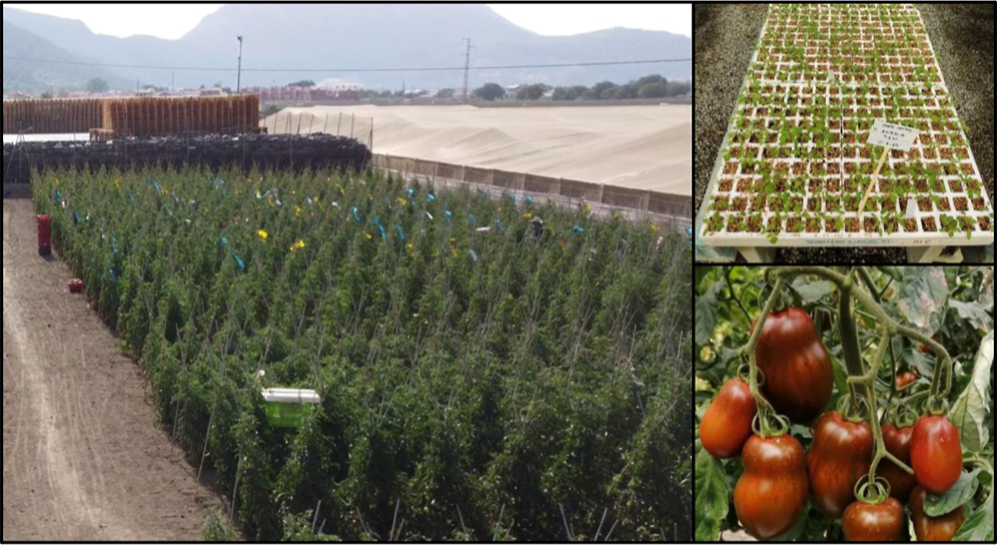
Traditional open-field tomato cultivation used in the present study (left). Tomato seedlings inoculated with the AM fungus *Rhizophagus irregularis* were prepared in commercial nurseries, where they were grafted and inoculated.

## 2 Materials and Methods

### 2.1 Plant and Microbial Material

Two large-scale open field experiments were performed in 2022 and 2023 in collaboration with a vegetable grower’s cooperative from Southern Spain, Hortoventas (https://www.hortoventas.com/) in Ventas de Zafarraya, Granada (Spain), following standard agricultural practices. Grafted plants were used for the experiments (Figure 2A). Tomato (*S. lycopersicum* × *Solanum habrochaites*) seeds of the rootstock Kardia (Syngenta, Spain) and the commercial variety Albenga (Unigen Seeds, Spain) were sown in 150-well starting trays with cell dimensions 3.5 × 3.5 × 6.5 cm (one seed per cell). Half of the seeds of rootstock Kardia were inoculated with 300 spores of the AM fungus *R. irregularis* (MUCL 57021) (Ri plants) supplied by Reka Soil (The Netherlands). The other half of the seeds were used as nonmycorrhizal controls (Nm plants). After 4 weeks of growing, scions of the tomato variety Albenga (Figure 2A) were grafted into the root-stock Kardia and maintained in the nursery for another 3 weeks. Finally, 7 week old tomato seedlings were transplanted into the field. For the two experiments, two different nurseries were used. In 2022, a conventional commercial nursery (San Isidro, Torrox, Spain) was used. Here, tomato seedlings were grown in starting trays containing a blond peat moss (Novarbo, Finland)–zeolite mixture (3:1). Standard management, including appropriate growing media and containers, regular irrigation and fertilization, and implementation of integrated pest and disease control, was used. In 2023, an organic commercial nursery (Saliplant, Puntalón, Spain) was used. Here, tomato seedlings were grown in starting trays containing nonsterile red peat-Wood 70:30 (v/v). The management used sustainable practices, such as the absence of chemical fertilizers and pesticides. In both years, a subset of plantlets (*n* = 6) was harvested before transplanting to the field (50 days after sowing, beginning of June) to quantify mycorrhizal colonization.

**Figure 2:**
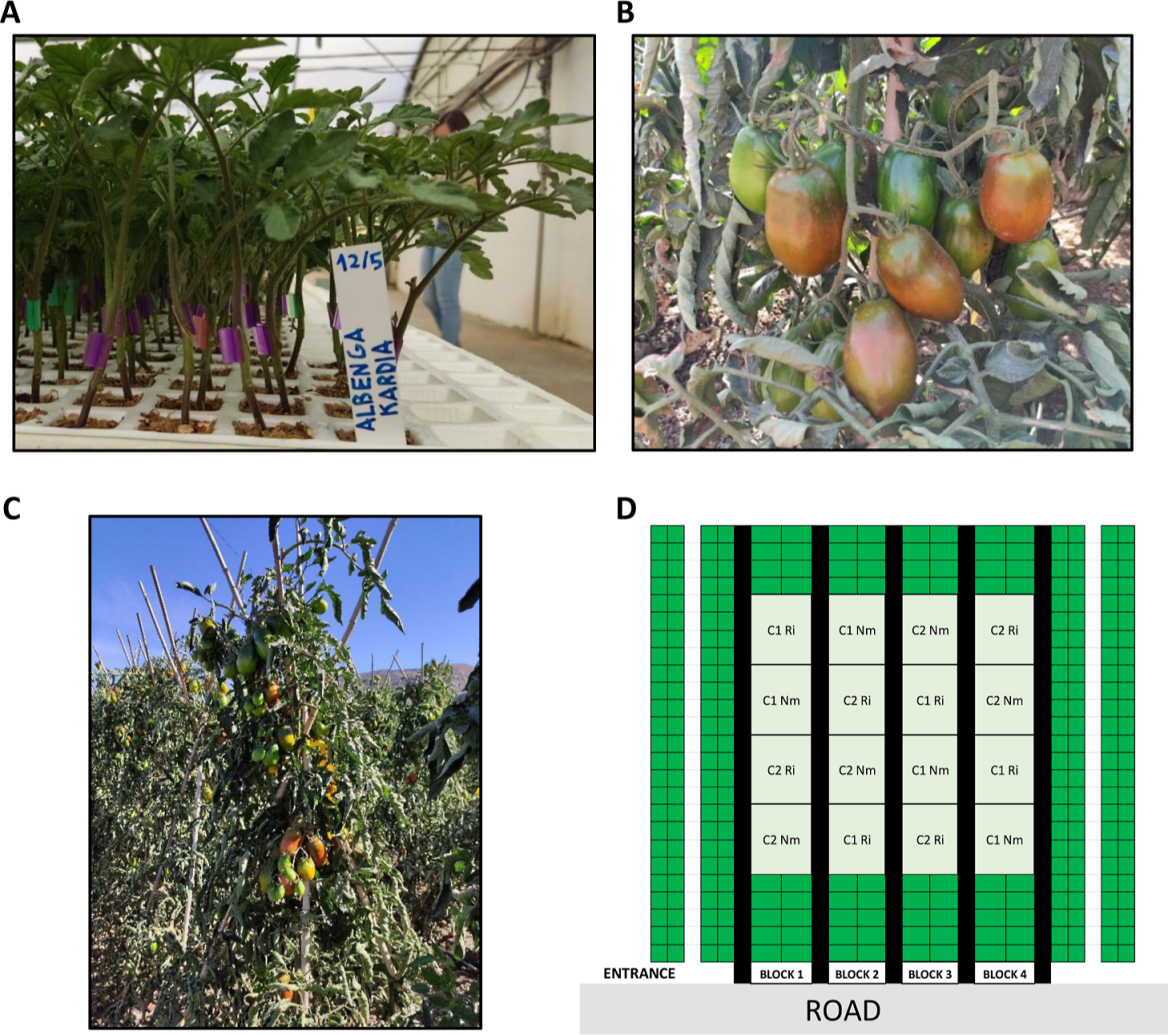
Tomato grafting and open-field experimental setup. Picture showing tomato grafted seedlings at the nursery (**A**). Tomato fruits from the variety Albenga (Unigen Seeds), a chocolate plum variety (**B**). Cultivation methodology with plants sown in “castle shape” (**C**). Experimental setup, following a randomized complete block design with four blocks (**D**).

### 2.2 Experimental Setup

For the two field experiments (2022 and 2023), the seedlings were transplanted into the experimental open-field plot from Hortoventas (SAT Hortoventas, Ventas de Zafarraya, Granada, Spain 36°57′26″N 4°07′14″W), and the plants were maintained during the whole crop cycle, from June to October. The field was a clayey soil consisting of calcareous fluvisols with 0.12% N, 3.10% total C, and 0.73% organic C. The mineral composition of the soil is described in Table S1. The climatological conditions in the area during the experiment are detailed in Table S2. The experimental field consisted of a traditional open field system with plants in “castle shape” of 45 m long and 25 m wide, with a total area of 1125 m^2^ (Figures 1 and 2C). Ri and Nm plants were organized by following a randomized complete block design with four blocks. Each block contained two sets for each treatment with 8 plants (pseudoreplicates) (*n* = 2 treatments × 2 replicates × 8 pseudoreplicates × 4 blocks = 128 plants) (Figure 2D). Tomato fruits were harvested in September of each season. Roots were harvested at the end of the season in October.

### 2.3 Quantification of Colonization Rates

Quantification of mycorrhizal colonization was performed by histochemical staining as previously described by García et al. (2020).^27^ Briefly, roots were harvested, cleared, and digested with 10% KOH (w/v) for 2 days at room temperature. The alkaline solution was removed, and the roots were washed thoroughly with tap water and acidified with a 2% (v/v) acetic acid solution. Then, the fungal structures were stained with a 5% (v/v) black ink (Lamy, Germany) and 2% acetic acid solution and incubated at room temperature for 24 h.^28^ The roots were washed with tap water to remove excess ink, and the colonization levels were determined by the gridline intersection method,^29^ using a Nikon SMZ1000 stereomicroscope. For colonization levels at the nursery stage, five replicates per treatment were analyzed. To quantify colonization levels in the field, root samples from six plants per block and treatment and three blocks were analyzed in 2022. In 2023, root samples from three plants per block and treatment and four blocks were analyzed.

### 2.4 Carotenoid Extraction from Tomato Fruits

Carotenoid extraction from tomato fruits was carried out as described by Stinco et al. (2013),^30^ with slight modifications. Fruits from five plants per block and for each treatment were analyzed. Three and four blocks were used in 2022 and 2023, respectively. 5 mg of the ground freeze-dried material was extracted with 0.5 mL of MeOH, 1 mL of chloroform and 0.5 mL of water into a Falcon tube. The samples were vortexed at 1500 × *g* for 5 min in an automatic vortex (Ohaus VXMTDG, OHAUS Europe GmbH, Nänikon, Switzerland) and then centrifuged at 4000*g* for 5 min at 4 °C. The organic colored fractions were collected separately and transferred to a clean tube, and the extraction process was repeated until an uncolored organic phase was obtained. The colored fractions from each sample were pooled, evaporated to dryness in a rotary evaporator (Eppendorf Concentrator Plus, Hamburg, Germany) at a temperature below 30 °C and stored until use under a nitrogen atmosphere.

### 2.5 Carotenoid Quantification by HPLC

For the HPLC analysis, each dried extract was dissolved in 200 μL of HPLC-grade ethyl acetate and centrifuged at 18000*g* for 5 min at 4 °C. Carotenoid analysis was carried out on an Agilent 1260 system (Agilent Technologies, Palo Alto, CA; USA), equipped with an UV/vis diode array detector, and using a C_30_ YMC column (3 μm, 150 × 4.6 mm) and a C_30_ YMC precolumn (2.7 μm, 50 mm × 4.6 mm) (Wilmington, NC, USA), as previously described by Stinco and co-workers.^30,31^ Methanol (MeOH) (solvent A), methyl-*tert*-butyl ether (MTBE) (solvent B), and water (H_2_O) (solvent C) were used in the mobile phase. Separation was achieved using the following gradient: 0 min 90% A + 5% B + 5% C, 0–5 min, 95% A + 5% B; 5–10 min, 89% A + 11% B, 10–16, min,75% A + 25% B; 16–20 min, 40% A + 60% B; 22.5–25 min, 15% A + 85% B; 25–28 min, and 90% A + 5% B + 5% C. The quantitative analysis of lycopene and β-carotene was made by external calibration. The tentative identification of the *Z*-isomers (*cis*-isomers) of lycopene [(9*Z*)-lycopene, (13*Z*)-lycopene, (15*Z*)-lycopene, and other (di-*Z*)-isomers of lycopene], the 9*Z*-isomer of β-carotene, and all-E β-carotene and lutein (all-E-lutein) was carried out by comparing their chromatographic and spectroscopic features with those of a standard mixture.^32^ Open lab ChemStation software was used, and the chromatograms were monitored at 285 nm for detection of phytoene, at 350 nm for phytofluene, at 450 nm for lutein and β-carotene, and at 472 nm for lycopene. Following the chromatographic validation criteria used by the authors in the methodological validation,^31^ the limits of detection and quantification (LOQ) were calculated from the calibration curves as 3 and 10 times the relative standard deviation of the analytical blank, respectively. The LODs ranged from 0.001 to 0.009 μg, and the LOQ ranged from 0.001 to 0.031 μg for lutein and β-carotene, respectively. The analyses were carried out in triplicate.

### 2.6 RNA Extractions and Gene Expression Analysis by RT-qPCR

Total RNA extraction and purification, synthesis of the corresponding cDNA, and qPCR were performed basically as described in Gamir et al. 2020.^33^ 10 mg of lyophilized tomato fruit from each pool was extracted using 1 mL of TRIsure reagent (Bioline, Barcelona, Spain) according to the manufacturer’s recommendations. Extracted RNA was treated with RQ1 DNase (Promega, Madrid, Spain) and purified through a silica column using the RNA Clean & Concentrator kit (Zymo Research, Madrid, Spain). RNA was quantified using a Nanodrop (Thermo Fisher Scientific, Madrid, Spain), and its integrity was checked by gel electrophoresis using E-Gel EX 2% Agarose gels (Invitrogen, Madrid, Spain). The first strand cDNA was synthesized with 1 μg of purified total RNA using the PrimeScript RT Master Mix kit (Takara Bio, Saint-Germain-en-Laye, France) according to the manufacturer’s instructions. Real-time quantitative PCR (qPCR) was performed in a StepOnePlus real-time PCR system (Thermo Fisher Scientific, Madrid, Spain), using the TB Green Premix ExTaq kit (Takara, Saint-Germain-en-Laye, France). Specific primers for carotenoid biosynthesis genes (Table S3) were used. Relative quantification of specific mRNA levels was performed using the comparative 2-Δ­(ΔCt) method.^34^ Expression values were normalized using the housekeeping gene *SlActin*2, encoding tomato actin (Table S3). Fruits from 5 plants per block and treatment and 4 blocks were analyzed.

### 2.7 Statistical Analysis

Statistical analyses were performed using R statistical language version 4.1.1.^35^ Graphical representations were produced with the ggplot2 package.^36^ The effect of AM fungal inoculation on the different response variables was analyzed using linear mixed effect models (lmer function from lmerTest package)^37^ with AM fungal treatments as the fixed factor and blocks as the random factor: lmer (variable ∼ treatment + (1|block)) (Table S4). Model validation was performed by checking the normality of the residuals using the shapiro.test function and the homogeneity of the variances using leveneTest function from the car package.^38^ If any of these assumptions was not met, data were log or square root transformed. The effect of AM fungal inoculation on the percentage of mycorrhizal colonization was analyzed using the nonparametric Kruskal–Wallis test (kruskal.test function) and differences between the different treatments were assessed with Dunn’s test using dunnTest function from the FSA package.^39^

## 3 Results and Discussion

Carotenoids are versatile isoprenoid compounds with important health-promoting properties for humans. These health benefits are not only associated with their antioxidant properties but also attributable to other mechanisms, such as light absorption or modulation of gene expression.^1,10^ A strategy to improve carotenoid content in foods is through biofortification,^2,7,40^ which is defined by the WHO as “the process by which the nutrient density of food crops is increased without sacrificing any characteristic that is preferred by consumers and/or farmers”. This can be achieved through plant breeding, biotechnology, or improved agronomic practices.^7^ Here, we assessed the impact of early inoculation with AM fungi as biostimulants on carotenoid content in tomato fruits as a sustainable strategy for biofortification in production systems. For that, two open-field trials in Southern Spain were conducted in consecutive years (2022 and 2023) under the standard agronomic practices of the tomato grower’s cooperative Hortoventas (Figure 1).

### 3.1 Inoculation with AM Fungi at the Nursery Phase Promotes Mycorrhizal Colonization

Healthy seedlings are an essential requirement for the production of high-quality plants and crops. Most vegetables are sown and grown in nurseries until they are ready to be transplanted to the field. This practice allows an efficient use of seeds to obtain uniform and vigorous plants, reduce the risk of pests and diseases, and achieve optimum growth of the root system. In the case of tomato, grafting in high vigor and pathogen resistant rootstocks is a major practice in intensive agriculture.^41^ Grafting is an increasingly popular horticultural technique and a standard practice in intensive vegetable production systems. This technique confers vigor and allows crops to be more resistant to soil pathogens and abiotic stresses, thus increasing the level of production. Typically, a scion of a vegetable species selected for its fruit quality characteristics is grafted onto a rootstock selected for its resistance and vigor. Tomato plants from the variety Albenga (Unigen Seeds), a chocolate plum variety, grafted into the rootstock Kardia (Syngenta, Spain) (Figure 2A,B) were inoculated with the AM fungus *R. irregularis* during the nursery stage. Before transplantation into the field, mycorrhizal root colonization levels were assessed in a subset of seedlings to confirm the successful establishment of AM symbiosis. In 2022, seedling generation and inoculation were carried out in a conventional nursery. Mycorrhizal colonization at the end of the nursery stage was low (about 3%) in inoculated (Ri) plants (Figure 3A, left panel). In 2023, an organic nursery was used and colonization levels in Ri plants were about 15% (Figure 3B, panel B). No colonization was detected in noninoculated (Nm) plants in either year at this stage (Figure 3A,B). The typical symbiotic structures, intraradical fungal hyphae (H) and arbuscules (Ar), were observed roots from inoculated plants (Ri-Nursery), indicating the establishment of a functional symbiosis (Figure 3C). As expected, these structures were absent in the Nm control plants (Figure 3C). The results show that early inoculation with AM fungi at the nursery stage is an efficient method to facilitate the contact between the host plant and the fungus and to obtain mycorrhizal plants. Moreover, better colonization rates were obtained when using an organic nursery. These types of nurseries are oriented toward sustainable production of vegetables and fruits. In addition to avoiding the use of synthetic fertilizers, pesticides, and fungicides, they use fewer inputs, conditions that favor plant–AM fungus interaction and the establishment of AM symbiosis.^42–44^ Therefore, early inoculation and the use of organic nurseries are preferable to ensure that the seedlings are primed and/or mycorrhized by the time they are transplanted to the field.

**Figure 3:**
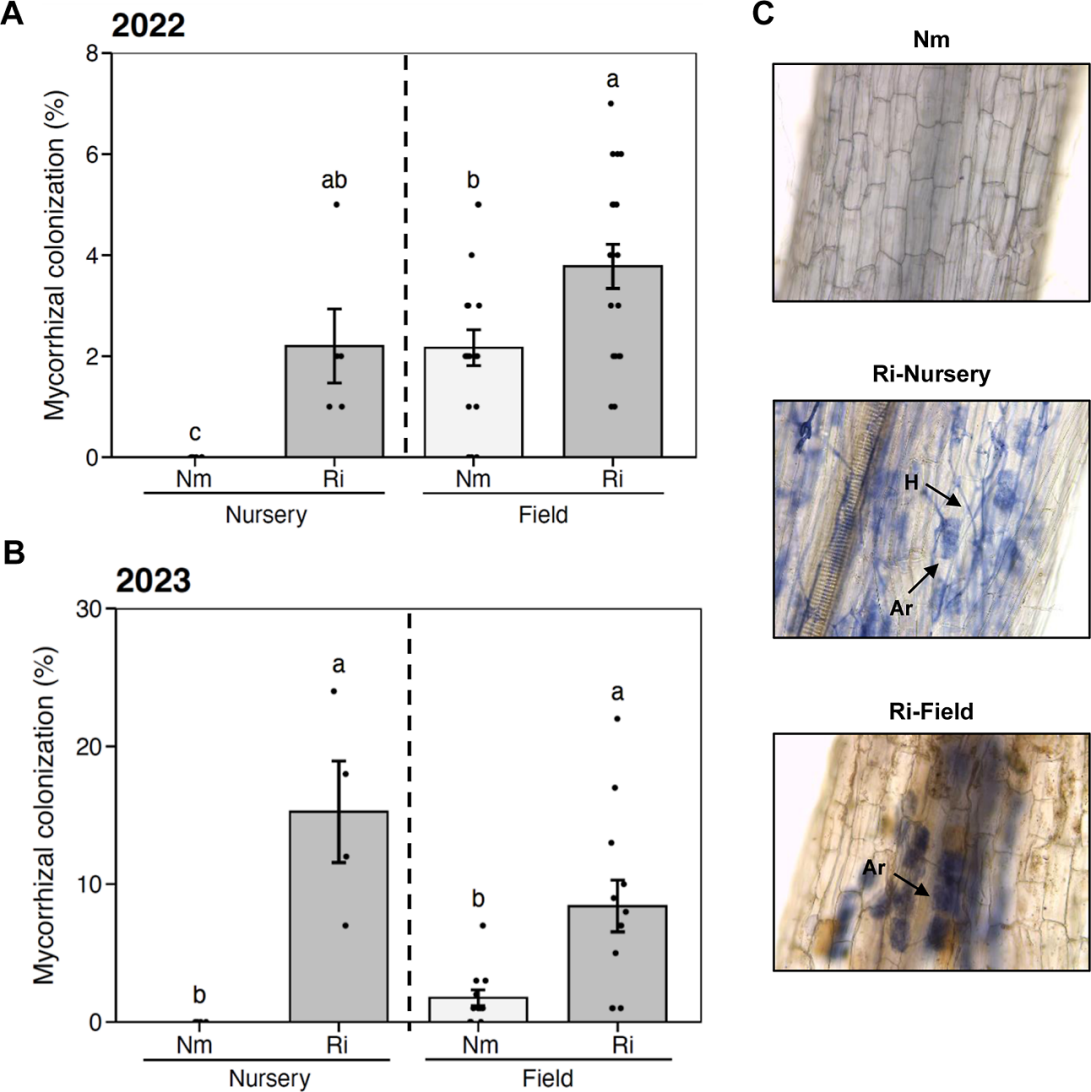
Mycorrhizal colonization levels in tomato roots. Colonization in the nursery stage and in open-field samples in two consecutive seasons, 2022 (**A**) and 2023 (**B**). (**C**) Right panel shows pictures of tomato root samples after black ink staining. Nm, root from mock inoculated 7 week-old seedlings; Ri-Nursery, root colonization by the AM fungus *Rhizophagus irregularis* at the nursery stage (7 week-old seedlings); Ri-Field, root colonization in plants harvested from the field experiment (5 month-old plants). Arrows indicate arbuscule (Ar) and intraradical hyphae (H). Bars represent the means, and error bars represent the standard error (±SE). Black dots represent the raw data. Data not sharing a letter in common differ significantly according to the Kruskal–Wallis test followed by Dunn’s posthoc test.

Mycorrhizal levels were also analyzed in roots harvested from the field at the end of the experimental trials. Here, AM colonization was observed in control Nm plants, likely due to the presence of resident AM fungi in the field soil (Figure 3A,B, right panel). However, significantly higher colonization was observed in Ri plants, with mycorrhization levels 2 and 3 times higher in 2022 (*p* = 0.023) and 2023 (*p* = 0.013), respectively (Figure 3A, B). At this stage, mature arbuscules were observed in the colonized roots (Figure 3C, Ri-Field). These results show that AM inoculation at the early nursery stages is effective and that the higher colonization levels with respect to noninoculated (Nm) plants are maintained when transplanted to the field. Remarkably, this type of inoculation presents a number of advantages over traditional inoculation methods at transplanting. For example, it requires fewer technical challenges since the biostimulant is applied at the time of seed sowing. Furthermore, growth conditions can be easier to control and adapt at this stage to facilitate interaction and the establishment of symbiosis. From an economic standpoint, this methodology would also be more advantageous. Since the host plant is smaller and more receptive at this nursery stage, it would require a smaller amount of inoculum.

### 3.2 Early AM Fungal Inoculation Improves Carotenoid Content in Tomato Fruits under Field Conditions

AM symbiosis can provide multiple benefits to the host plant, including improved nutrition, enhanced tolerance against abiotic stresses (drought, salinity, heat stress, etc.), and increased resistance to pathogens and pest without incurring yield losses.^16,17^ In our field trials, preinoculation with *R. irregularis* during the nursery stage did not significantly impact plant height and number of flowers, fruit yield, weight nor size (Table S5). We also evaluated the effect of early AM fungal inoculation on tomato quality. For that, the carotenoid content of tomato fruits from inoculated and noninoculated plants was analyzed. Tomatoes are an important source of carotenoids that contribute to human health.^9,10,12^ Lycopene is the main carotenoid present in ripe tomato fruits, being responsible for their characteristic red color. Other colored carotenoids, such as β-carotene and lutein are present, although in smaller quantities.^11^ Tomato fruits are also a source of the colorless carotenoids phytoene and phytofluene. These carotenoids have been shown to be major dietary carotenoids, and there is increasing evidence suggesting they could be involved in health-promoting biological actions.^13^ Although a decrease of *z*-phytoene was found in fruits from Ri plants in 2022 (*p* = 0.007, Table 1), no major changes were observed in phytoene and phytofluene content in fruits from inoculated and noninoculated plants from any of the growing seasons (2022 and 2023) (Tables 1 and 2). Similarly, the levels of the colored carotenoid lutein remained unaltered by the AM treatment in any of the years analyzed (Tables 1 and 2). Conversely, a clear effect of AM fungal inoculation was observed on lycopene and β-carotene content. In the 2022 campaign, a significant increase was detected in a *Z*-lycopene isomer (*p* = 0.003), while no differences were observed for the other isoforms analyzed [(5*Z*)-, (9*Z*)-, and (15*Z*)-lycopene] (Table 1). Overall, an increase of about 30% in total lycopene was detected in tomato fruits from plants preinoculated with *R. irregularis* (*p* = 0.046, Figure 4A). Regarding β-carotene, a promotion on the content of the isomers 9*Z* (*p* = 0.025) and all-*E*-β-carotene (*p* = 0.01) was observed in inoculated plants (Table 1), with a total increase in β-carotene content of about 40% ((*p* = 0.007, Figure 4B). In 2023, a promotion in *Z*-lycopene (*p* = 0.0002) was also observed in preinoculated plants, with a total increase in lycopene of about 45% (*p* = 0.048, Table 2 and Figure 4C). The levels of all-*E*-β-carotene (*p* = 0.008) were also higher in fruits from inoculated plants, with a total increase in β-carotene of about 45% (*p* = 0.008, Table 2 and Figure 4D). Different total levels/contents of carotenoids were observed in 2022 and 2023. These differences might be due to different factors, such as environmental conditions, as carotenoids are highly influenced by light and temperature,^45^ or to the extraction process for the analysis. In any case, the relative contents of lycopene and β-carotene were consistently higher in plants early inoculated with *R. irregularis* (Ri) compared to noninoculated (Nm) plants in both years.

**Table 1:** Colorless and Colored Carotenoid Content in Tomato Fruits (Field Experiment 2022)^a^

	colorless carotenoid content (μg/g fruit)	
carotenoid	Nm	Ri
*Z*-phytoene	0.28 ± 0.03	0.19 ± 0.02**
(15*Z*)-phytoene	2.67 ± 0.24	2.83 ± 0.20
total phytoene	2.94 ± 0.26	3.02 ± 0.21
*Z*-phytofluene 1	0.63 ± 0.04	0.68 ± 0.07
*Z*-phytofluene 2	0.51 ± 0.03	0.58 ± 0.03
total *Z*-phytofluene	1.14 ± 0.07	1.26 ± 0.10

carotenoid	colored carotenoid content (μg/g fruit)	
	Nm	Ri
*Z*-lycopene	3.13 ± 0.25	4.44 ± 0.25**
(9*Z*)-lycopene	1.91 ± 0.13	2.28 ± 0.18
(13*Z*)-lycopene	2.78 ± 0.26	3.40 ± 0.32
(15*Z*)-lycopene	0.61 ± 0.09	0.54 ± 0.04
*trans*-lycopene	113.42 ± 8.64	139.43 ± 9.83*
All-*E*-β-carotene	4.30 ± 0.26	6.06 ± 0.40*
(9*Z*)-β-carotene	0.26 ± 0.02	0.37 ± 0.04*
Lutein	1.33 ± 0.11	1.34 ± 0.13

a Upper panel, analysis of colorless carotenoid content by HPLC. The levels of different isomers of phytoene and phytofluene were analyzed. Lower panel, analysis of colored carotenoids. The levels of different isomers of lycopene, β-carotene, and lutein were analyzed. Data represent the means of 16 independent replicates, four biological replicates per four blocks (±SE). Asterisks indicate statistically significant (*p < 0.05, **p < 0.01, ***p < 0.001) differences compared to control Nm treatment.

**Table 2:** Colorless and Colored Carotenoid Content in Tomato Fruits (Field Experiment 2023)^a^

carotenoid	colorless carotenoid content (μg/g fruit)	
	Nm	Ri
*Z*-phytoene	0.28 ± 0.04	0.26 ± 0.03
(15*Z*)-phytoene	5.37 ± 0.38	5.09 ± 0.42
total phytoene	5.65 ± 0.39	5.35 ± 0.44
*Z*-phytofluene 1	1.05 ± 0.11	1.16 ± 0.11
*Z*-phytofluene 2	3.35 ± 0.24	3.15 ± 0.26
total *Z*-phytofluene	4.38 ± 0.35	4.31 ± 0.36

carotenoid	colored carotenoid content (μg/g fruit)	
	Nm	Ri
*Z*-lycopene	2.02 ± 0.19	3.14 ± 0.24***
(9*Z*)-lycopene	0.11 ± 0.02	0.14 ± 0.02
(13*Z*)-lycopene	0.21 ± 0.02	0.26 ± 0.03
(15*Z*)-lycopene	0.35 ± 0.02	0.34 ± 0.03
*trans*-lycopene	14.25 ± 1.42	20.35 ± 1.26*
All-*E*-β-carotene	1.30 ± 0.09	1.85 ± 0.12**
(9*Z*)-β-carotene	0.14 ± 0.02	0.18 ± 0.02
Lutein	0.90 ± 0.09	1.04 ± 0.10

a Legend as indicated in Table 1.

**Figure 4:**
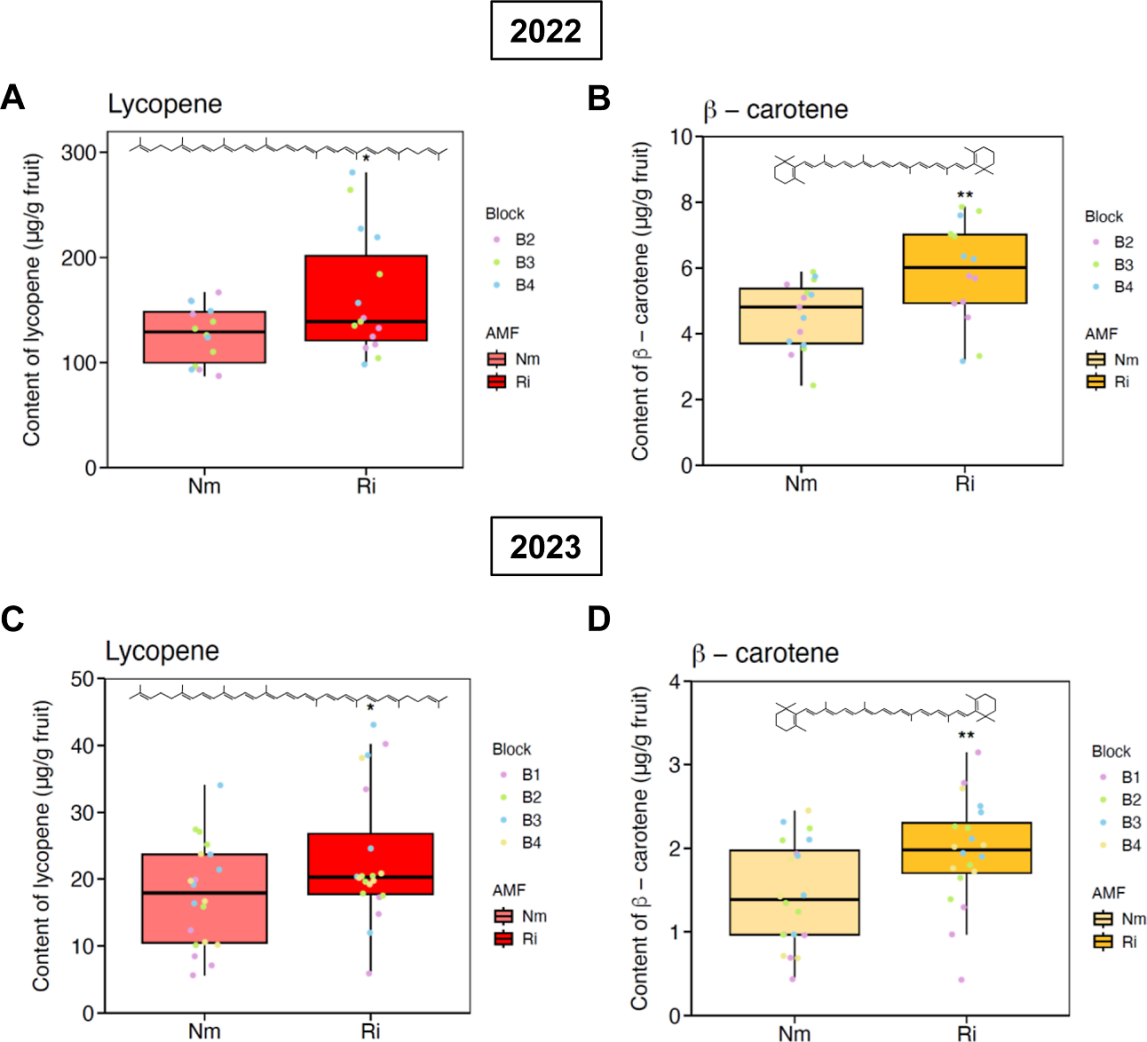
Carotenoid content in field-grown tomato fruits in two consecutive seasons. Total content on lycopene (**A** and **C**) and β-carotene (**B** and **D**) in ripe tomato fruits harvested in summer 2022 and 2023. Plants were inoculated with the AM fungus *Rhizophagus irregularis* (Ri). Noninoculated plants (Nm) were included as a control. Boxes represent the interquartile range, black lines indicate the median, whiskers represent the maximum and minimum within 1.5 times the interquartile range, and colored dots represent the raw data. B1–B4 correspond to samples from different blocks from the experiments. Asterisks indicate statistically significant (*p < 0.05, **p < 0.01) differences compared to control Nm treatment, according to linear mixed effect models.

A similar effect of AM fungal inoculation on the tomato fruit carotenoid content has been previously shown under greenhouse conditions in a few studies. Ullah and co-workers showed that inoculation with the AM fungus *Funneliformis mosseae* increased lycopene and β-carotene levels about 48 and 30%, respectively, without compromising fruit yield.^20^ A similar trend, although not significant, was observed for these two carotenoids in tomato plants inoculated with *R. irregularis*.^19^ In another experiment, it was shown that inoculation with the AM fungi *R. irregularis* and *F. mosseae* increased lycopene levels about 12 and 10%, respectively, while no changes in β-carotene were observed.^18^ Notably, in all these three experiments, plants were grown in pots and inoculated with the AM fungi during the early stages of development.^18–20^ These results under experimental field conditions show that early inoculation with AM fungi can significantly improve tomato fruit quality and can be used as a carotenoid biofortification strategy. This increase in the level of bioactive carotenoids is similar to those obtained using other carotenoid biofortification strategies. For example, overexpression of the *PSY1* gene, encoding a key enzyme in carotenoid biosynthesis in fruits,^9^ resulted in an increase of 25% in the total carotenoid content of ripe fruits.^46^ A more detailed analysis showed an increase of about 40% in β-carotene, while no significant changes were observed in lycopene content.^46^ A recent analysis of carotenoid content in fruit from varieties marketed as ‘high lycopene’ tomatoes showed that 19 out of 42 genotypes had at least two times more lycopene content than the reference genotype MoneyMaker.^47^ Conversely, β-carotene levels were generally reduced in most genotypes. In contrast, we show here that early AM fungal inoculation is a good strategy to improve both lycopene and β-carotene levels in tomato fruits.

Our results also show that AM biofortification effects are graft transmissible from the colonized hybrid rootstock Kardia (*S. lycopersicum* × *S. habrochaites*) to the *S. lycopersicum* commercial variety scion Albenga. Grafting is an increasingly horticulture technique, which confers vigor and improves crop tolerance and resistance.^41^ Thus, the rootstock can be inoculated early during the nursery stage, and then the benefits of the AM symbiosis, including the carotenoid content in fruit, can be transmitted to the variety of interest. This opens up a wide range of possibilities for improving the organoleptic quality of the fruits. There is a great diversity of tomatoes on the market, with thousands of varieties depending on interests and needs, color, flavor, size, and shape.^48^ The methodology that we propose here could be applied to the vast majority of varieties without having to develop new improved varieties. However, further studies are needed to determine the extent of these benefits. As already mentioned, lycopene and β-carotene are very important carotenoids in nutrition and health promotion.^7,9,12^ Because of these health benefits, there is great interest in producing lycopene- and β-carotene-rich products, the so-called “functional foods”. Here, we demonstrate that early inoculation of rootstocks with AM fungi at the nursery stage is an efficient method to facilitate plant–fungus interactions and promote AM symbiosis. Also, this is effective for the graft transmissible biofortification of carotenoids in tomato fruits under production conditions.

### 3.3 AM Fungal Inoculation Promotes Carotenoid Biosynthesis in Ripe Fruits

In order to explore the mechanisms underlying this biofortification, we analyzed the transcriptional regulation of the carotenoid biosynthesis in fruits. It is known that AM symbiosis impacts carotenoid metabolism in roots in order to modulate the production of the apocarotenoids strigolactones, mycorradicin, α-ionols, and blumenols. These carotenoid-derived compounds are important to regulate both the establishment and maintenance of a functional symbiosis.^49,50^ However, whether AM symbiosis also impacts carotenoid biosynthesis in fruits remains largely unexplored. Here, we explore the impact of early AM fungal inoculation on the carotenoid metabolism of fruits by analyzing the expression of several key carotenoid biosynthesis genes by real-time quantitative PCR (RT-qPCR) in the fruits from the 2023 campaign. The expression levels of the central genes involved in the major steps of carotenoid biosynthesis (*SlDXS1*, encoding for 1-deoxy-*d*-xylulose 5-phosphate synthase 1; *SlGGPPS1*–*3*, geranylgeranyl pyrophosphate synthase 1, 2, and 3; *SlPSY1*, PSY 1; *SlPDS*, PDS; *SlZDS*, z-carotene desaturase; *SlCrtISO*, carotenoid isomerase; and *SlLYC*-*B*, encoding for lycopene β-cyclase) (Figure 5A) were assessed in tomato fruits from plants inoculated with *R. irregularis* (Ri) or noninoculated plants (Nm) (Figure 5B).

**Figure 5:**
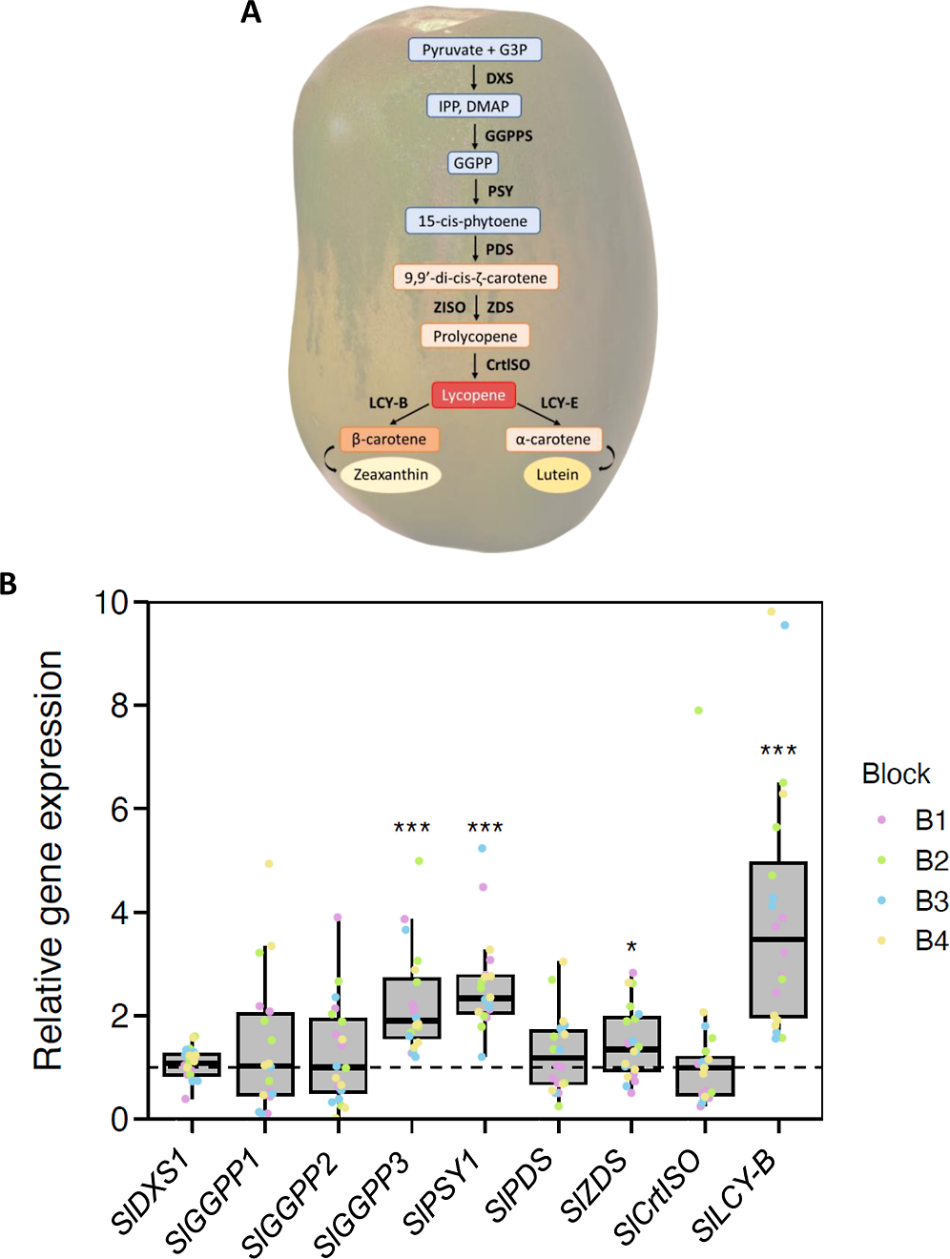
Carotenoid biosynthesis pathway and gene expression analysis in ripe tomato fruits. (**A**) Rectangular boxes represent the substrates involved in carotenoid biosynthesis. The carotenoid biosynthesis enzymes located in the plastids are indicated in bold. DXS, 1-deoxy-*d*-xylulose 5-phosphate synthase; GGPPS, geranylgeranyl pyrophosphate synthase; PSY1, PSY 1; PDS, phytoene desaturase; ZISO, ζ-carotene isomerase; ZDS, ζ-carotene desaturase; CrtISO, carotene isomerase; LCY-B, lycopene β-cyclase; LCY-E, lycopene ε-cyclase. (**B**) Gene expression analysis (fold-change) by qPCR of carotenoid biosynthesis genes in fruits from noninoculated plants (Nm) and inoculated with *R. irregularis* (Ri). Expression values were normalized with the housekeeping gene *SlActin2*. The dashed line represents the relative gene expression of Nm fruits. Boxplots represent the relative gene expression of Ri fruits, where boxes represent the interquartile range, black lines indicate the median, whiskers represent the maximum and minimum within 1.5 times the interquartile range, and colored dots represent raw data. B1–B4 correspond to samples from different blocks from the experiment. Asterisks indicate statistically significant (*p < 0.05, **p < 0.01, ***p < 0.001) differences compared to control Nm treatment, according to linear mixed effect models.

No significant changes were detected in the expression of the genes *SlDXS1*, *SlGGPPS1*–*2*, *SlPDS*, *SlCrtISO*, and *SlZDS* between the two treatments (Figure 5B). However, an increase of about 2-fold was observed in the expression of *SlGGPS3* in fruits from Ri plants compared to control Nm plants (*p* = 1.6 × 10^–9^, Figure 5B). GGPPSs produce GGPP, which serves as a precursor for many plastidial isoprenoids, including carotenoids. In tomato, three GGPPS isoforms, SlGGPPS1, SlGGPPS2, and SlGGPPS3, have been characterized. Of these, SlGGPPS2 and SlGGPPS3 have been shown to be expressed in the fruit pericarp and to be related to carotenoid biosynthesis in fruits. Barja and co-workers also showed that SlGGPPS3-deficient mutant plants had a strong reduction in phytoene and lycopene levels, indicating that this enzyme is key in the biosynthesis of carotenoids in fruits.^51^ Our results support the role of this enzyme in carotenoid biosynthesis in ripe tomato fruits and show that its expression is promoted in fruits from AM inoculated plants. An induction of about 2.5-fold was also detected in the expression of *SlPSY1* in fruits from Ri plants (*p* < 2 × 10^–9^, Figure 5B). PYS converts GGPP into phytoene, which is the first committed intermediate of the carotenoid pathway (Figure 5A), being a paramount enzyme of carotenogenesis during tomato fruit ripening.^9^ Indeed, PSY is a target for genetic engineering biofortification strategies. Plants showing a constitutive expression of PSY genes in tomato resulted in increased levels of carotenoids in different tissues.^9^ In tomato, there are three PSY isoforms, SlPSY1, SlPSY2, and SlPSY3, regulating carotenoid biosynthesis in different tissues.^51^ SlPSY3 is mainly expressed in roots and is related to the biosynthesis of apocarotenoids (strigolactones, mycorradicin, and α-ionols) in mycorrhizal plants.^50,52^ SlPSY2 is mainly present in photosynthetic tissues and associated with carotenoids involved in photosynthesis and photoprotection.^53^ Finally, SlPSY1 is mainly present in fruits, and its expression is promoted during ripening to produce carotenoids associated with fruit pigmentation.^51,53^ We confirm these results and show that *SlPSY1* expression is further promoted by AM symbiosis. PSYs form a multienzymatic complex with other enzymes of the carotenogenic pathway, including the isopentenyl diphosphate isomerase and GGPPSs.^52,54^ Interestingly, it was shown that SlPSY1 interacts with SlGGPP3 in tomato fruits to support carotenoid biosynthesis for fruit pigmentation.^51^ Our results are in line with this observation and show that the tandem SlGGPP3–SlPSY1 is induced in tomato fruits from preinoculated mycorrhizal plants to promote the production of β-carotene and lycopene.

We also observed a 3.4-fold increase in the expression of the gene *SlLYC*-*B* in Ri plants compared to control Nm plants (*p* < 2 × 10^–9^, Figure 5B). LCY is another key enzyme in carotenogenesis. This enzyme catalyzes the cyclization of lycopene to produce carotenoids with α or β rings. There are two LCY enzymes, LCY-E and LCY-B, which produce α-carotene and β-carotene, respectively (Figure 5A).^9^ It was shown that constitutive expression of the carrot *LCY*-*B1* gene in tobacco increased the content in β-carotene and total carotenoids.^55^ Similarly, the overexpression of *SlLCY*-*B* in tomato increased the levels of β-carotene in ripe fruits while decreasing the lycopene content.^56,57^ Conversely, it was shown that the null mutation of this gene resulted in a huge reduction of β-carotene and an increase in lycopene.^56^ Therefore, the induction of *SlLCY*-*B* we observed in Ri plants would be responsible for the increase in β-carotene in tomato fruits. Remarkably, in our study lycopene levels remained high too, probably as a consequence of the induction of key genes in previous steps of the biosynthetic pathway.^9,35^ Our gene expression data confirm transcriptional activation of the carotenoid pathway at multiple key steps in grafted plants inoculated with AM fungi, supporting the activation of secondary metabolism by the early establishment of symbiosis and/or plant–AM fungal interaction. We cannot rule out that other mechanisms, such as post-transcriptional regulation or modulation of enzymatic activity, may also contribute to the observed increase.^7,40^ Remarkably, this early activation or priming of the carotenoid pathway was graft transmissible to fruits. Thus, we show here that this is a feasible strategy to improve carotenoid biofortification in fruits. It would be interesting to investigate how this activation is regulated. AM symbiosis is known to have a significant hormonal impact on the host plant, both locally in the roots and systemically in the leaves and fruits.^16,58^ Remarkably, the biosynthesis of carotenoids is regulated by the carotenoid’ derived hormones abscisic acid and strigolactones, which are modulated in the roots during AM symbiosis.^49,50,59^ Whether these hormones regulate, either directly or indirectly, the biosynthesis of carotenoids in distal tissues, such as fruits, remains to be explored. It should be noted that other current strategies to increase carotenoid accumulation are focused on reducing their degradation, or enhancing their storage and retention in plant tissues.^40^ Whether these mechanisms are also modulated in inoculated or mycorrhizal plants is an open question.

Overall, we show here that early inoculation of tomato plants with AM fungi during the nursery stage is a suitable and efficient method to facilitate host–plant–fungus interaction and to promote symbiosis establishment. We also demonstrate for the first time that the effects of early inoculation of rootstocks with AM fungi are graft transmissible and are maintained under field conditions, providing plants with long-lasting benefits. Among them is the promotion of carotenoid biosynthesis in fruits, resulting in an increase in lycopene and β-carotene content. Thus, AM fungi can be used as a strategy for carotenoid biofortification in tomato production systems. Since most agricultural crops are mycorrhizal^15^ and predeveloped in nurseries, it would be interesting to assess whether these health promoting properties also occur in other plant species. This will help to produce biofortified “functional products” by implementing the use of AM fungi as biostimulants in modern and sustainable agriculture. Increasing the carotenoid content is only the first step in the biofortification pathway. Other important steps, such as stability during postharvest and bioaccessibility, must be taken into account.^7^ In this regard, further efforts should be directed toward these steps to ensure that the increase in carotenoids results in improved intake and greater health benefits.

## Supplementary Material





## Data Availability

The data sets and material generated during and/or analyzed during the current study are available from the corresponding author on reasonable request.

## Notes

Table S1. Nutrient content in soil from the cooperative Hortoventas; Table S2. Climatological data in the cooperative Hortoventas (Ventas de Zafarraya, Granada, Spain) during the years of the open-field experiments (2022 and 2023); Table S3. Primer sequences for the carotenoid biosynthesis genes used in the real-time qPCR analysis; Table S4. Detailed information on the statistical analyses performed on each of the measured variables; and Table S5. Physiological parameters of tomato plants grown and fruits in the field during the seasons 2022 and 2023 (PDF)
Compiled raw data (XLSX)

## Abbreviations

AM: arbuscular mycorrhizal
PGPR: plant growth promoting rhizobacteria
PSY: phytoene synthase
PDS: phytoene desaturase
CrtISO: carotene isomerase
ZDS: ζ-carotene desaturase
LCY-B: lycopene β-cyclase
LCY-E: lycopene ε-cyclase
RT-qPCR: real-time quantitative PCR
DXS: 1-deoxy-*d*-xylulose 5-phosphate synthase
GGPPS: geranylgeranyl pyrophosphate synthase
