# Supplementary material for: Carotenoid Biofortification in Field-Grown Tomato Fruits by Early Inoculation with Arbuscular Mycorrhizal Fungi
Source: J Agric Food Chem. 2025 Dec 10;73(51):32612–22. doi: 10.1021/acs.jafc.5c14198 (PMC12751021; doi:10.1021/acs.jafc.5c14198)
Supplement: Supplementary file 1 [file jf5c14198_si_001.pdf]

Supporting information for:

Carotenoid biofortification in field-grown tomato fruits by early inoculation with arbuscular mycorrhizal fungi

Javier Lidoy[1†], Zhivko Minchev[1,2†], Luis España-Luque[1], Ana M. Benítez-González[3], Andrea Ramos[1], Juan García[1], Estefanía Berrio[1], Olena Nesterenko[1], Pedro Díaz-Ortiz[4], Antonio J. Meléndez-Martínez[3], María J. Pozo[1], Juan A. López-Ráez[1*]

Table S1. Nutrient content in soil. Values represent the means of 3 independent and randomized soil replicates (±SE).

Nutrient content (mg/Kg)		Nutrient content (mg/Kg)		Nutrient content (mg/Kg)	
Ion		Ion		Ion	
**Al**	46114.49 ± 1958.02	**Fe**	28363.24 ± 1122.68	**Pb**	17.80 ± 1.05
**As**	8.32 ± 0.16	**K**	12879.88 ± 451.06	**S**	232.01 ± 7.08
**Ca**	32130.05 ± 1964.71	**Li**	37.26 ± 1.63	**Si**	6007.26 ± 208.83
**Cd**	1.68 ± 0.56	**Mn**	536.02 ± 28.28	**Sr**	79.13 ± 3.57
**Cr**	54.48 ± 2.38	**Na**	484.56 ± 20.17	**Ti**	1269.31 ± 21.73
**Mg**	20626.88 ± 468.32	**Ni**	29.88 ± 1.28	**V**	72.10 ± 2.23
**Cu**	25.32 ± 2.45	**P**	65.08 ± 2.72	**Zn**	75.18 ± 6.20

Table S3. Primer sequences used in the real time qRT-PCR analysis.

Gene	ID	Primers (5'-3')
SlDXS1[1]	Solyc01g067890	TGACCATGGATCTCCTGTTG<br>GCCTCTCTGGTTTGTCCAAG
SlGGPPS1[2]	Solyc11g011240	GGCCTTTGAACATGTGGCTACC<br>ACTCGCCAAGTCCACAATTTGC
SlGGPPS2[2]	Solyc04g079960	AAAGTCATCGTCGGAGCTCG<br>GTTTAGCTTCGCCGTTGAGC
SlGGPPS3[2]	Solyc02g085700	AGGAGGTGCACCAGATGAAG<br>TCAGCAACCAAGTCCTTCCC
SlPSY1[1]	Solyc03g031860	GCCATTGTTGAAAGAGAGGGTG<br>AGGCAAACCAACTTTTCCTCAC
SlPDS[1]	Solyc03g123760	AGCAACGCTTTTTCCTGATG<br>TCGGAGTTTTGACAACATGG
SlZDS[3]	Solyc01g097810	GCTGCTGAGTTGAATGACATCTC<br>GTGCGATGCCTAACTGAGTTG
SlCrtISO[4]	Solyc10g081650	TTTTGGCGGAATCAACTACC<br>GAAAGCTTCACTCCCACAGC
SlLYC-B[1]	Solyc10g079480	TTGTGGCCCATAGAAAGGAG<br>GGCATCGAAAAACCTTCTTG
SlEF-1[5]	Solyc06g009960	GATTGGTGGTATTGGAACTGTC<br>AGCTTCGTGGTGCATCTC
SlActin2[6]	Solyc11g005330	TTGCTGACCGTATGAGCAAG<br>GGACAATGGATGGACCAGAC

[1]Briardo-Llorente *et al.*, 2016; [2]Barja *et al.,* 2021; [3]McQuinn *et al.*, 2020; [4]Enfissi *et al.,* 2017; [5]Rotenberg *et al.*, 2006; [6]Yan *et al.*, 2013.

Table S4. Detailed information on the statistical analyses performed on each of the measured variables. Asterisks indicate significance level: *$p < 0.05$, **$p < 0.01$, ***$p < 0.001$.

					Year					
					2022			2023		
**Variable**	**Function**	**Family**	**Random**	**Df**	**N**	**Chisq**	**Pr(>Chisq)**	**N**	**Chisq**	**Pr(>Chisq)**
Z-phytoene	lmer	normal	(1|block)	1	30	7.2060	0.0073 **	40	0.4098	0.5221
(15*Z*)-phytoene	lmer	normal	(1|block)	1	30	0.5505	0.4581	40	0.5109	0.4748
Total phytoene	lmer	normal	(1|block)	1	30	0.1628	0.6866	40	0.5434	0.461
Z-phytofluene 1	lmer	normal	(1|block)	1	30	0.5855	0.4442	40	0.2836	0.5944
Z-phytofluene 2	lmer	normal	(1|block)	1	30	2.7270	0.0987	40	0.2930	0.5883
Total Z-phytofluene	lmer	normal	(1|block)	1	30	1.5507	0.213	40	0.0320	0.8579
Z-lycopene	lmer	normal	(1|block)	1	30	9.0679	0.0026 **	40	13.665	0.0002 ***
(9*Z*)-lycopene	lmer	normal	(1|block)	1	30	0.5902	0.4424	40	2.2630	0.1325
(13*Z*)-lycopene	lmer	normal	(1|block)	1	30	0.7302	0.3928	40	2.3500	0.1253
(15*Z*)-lycopene	lmer	normal	(1|block)	1	30	0.4659	0.4949	40	0.0740	0.7856
Trans-lycopene	lmer	normal	(1|block)	1	30	2.6711	0.1022	40	2.6593	0.1029
Total lycopene	lmer	normal	(1|block)	1	29	4.002	0.0455 *	40	3.921	0.0477 *
All-*E*-β-carotene	lmer	normal	(1|block)	1	29	6.5048	0.0108 *	40	7.1344	0.0076 **
(9*Z*)-β-carotene	lmer	normal	(1|block)	1	29	4.9909	0.0255 *	40	1.5976	0.2062
Total β-carotene	lmer	normal	(1|block)	1	29	7.2633	0.007 **	40	6.995	0.0082 **
Lutein	lmer	normal	(1|block)	1	29	0.0003	0.9853	40	1.1032	0.2936
SlDXS1	lmer	normal	(1|block)	1				40	0.0115	0.9475
SlGGPP1	lmer	normal	(1|block)	1				40	0.4970	0.4808
SlGGPP2	lmer	normal	(1|block)	1				40	0.0002	0.9888
SlGGPP3	lmer	normal	(1|block)	1				40	36.369	1.6e-09 ***
SlPSY1	lmer	normal	(1|block)	1				40	92.213	< 2e-16 ***
SlPDS	lmer	normal	(1|block)	1				40	0.1756	0.6752
SlZDS	lmer	normal	(1|block)	1				40	4.8664	0.02738 *
SlCrtISO	lmer	normal	(1|block)	1				40	0.6479	0.4209
SlLCY-B	lmer	normal	(1|block)	1				40	96.658	< 2e-16 ***
Mycorrhizal colonization	kruskal.test			3	46	17.6	0.0005 ***	32	19.391	0.0002 ***

Table S5. Physiological parameters from tomato plants grown under field conditions.

Physiological parameter	Treatment			
	2022		2023	
	Nm	Ri	Nm	Ri
Plant height (m)	1.61 ± 0.01	1.67 ± 0.02*	1.66 ± 0.02	1.72 ± 0.04
Bunches of flowers	4.41 ± 0.11	4.67 ± 0.12	5.01 ± 0.15	4.78 ± 0.22
Fruit yield (kg)	0.69 ± 0.11	0.75 ± 0.09	1.12 ± 0.28	0.96 ± 0.33
Fruit weight (g)	107.96 ± 3.36	114.67 ± 3.67	111.21 ± 5.43	115.86 ± 4.04
Fruit size (mm)	51.96 ± 0.62	52.63 ± 0.73	52.29 ± 0.29	50.87 ± 0.88
